# Supplementary figures and images for: Predictive performance of genomic selection methods for carcass traits in Hanwoo beef cattle: impacts of the genetic architecture
Source: Genet Sel Evol. 2017 Jan 4;49:1. doi: 10.1186/s12711-016-0283-0 (PMC5240470; doi:10.1186/s12711-016-0283-0)

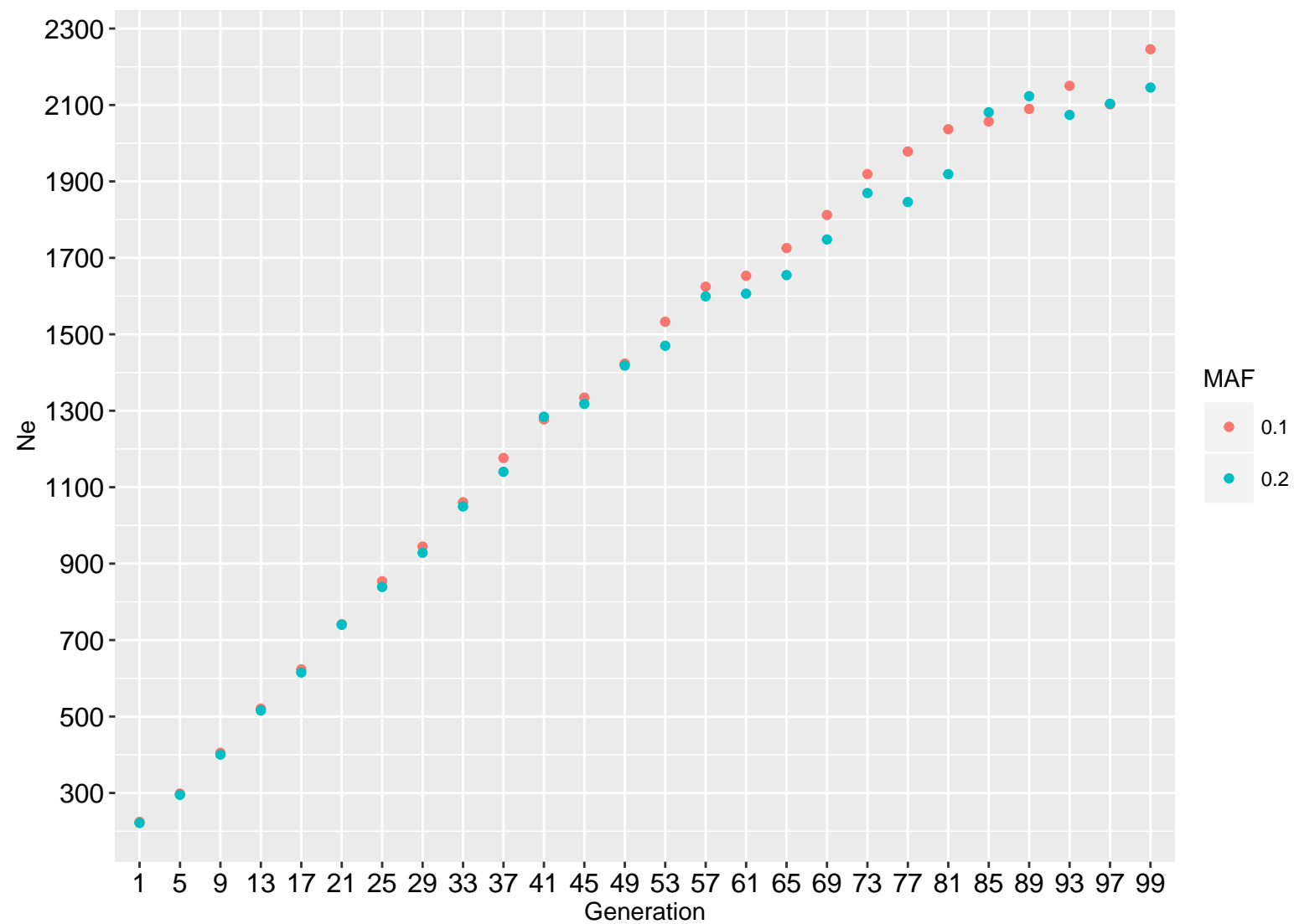

Supplement: Supplementary file 1 — Additional file 1: Figure S1. Estimates of effective population size ($N_{e}$Ne) in the past generations. Thresholds of 0.1 and 0.2 were considered for minor allelic frequency (MAF). The figure describes the changes of N e over generations for two different minor allelic frequencies (0.1 and 0.2). [file 12711_2016_283_MOESM1_ESM.pdf]

**BT**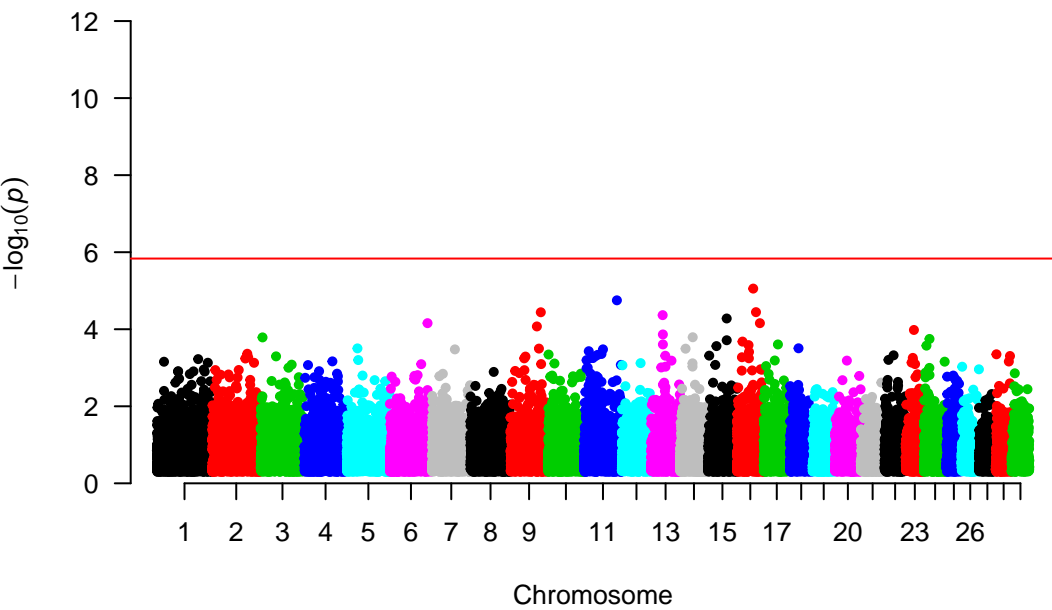**CW**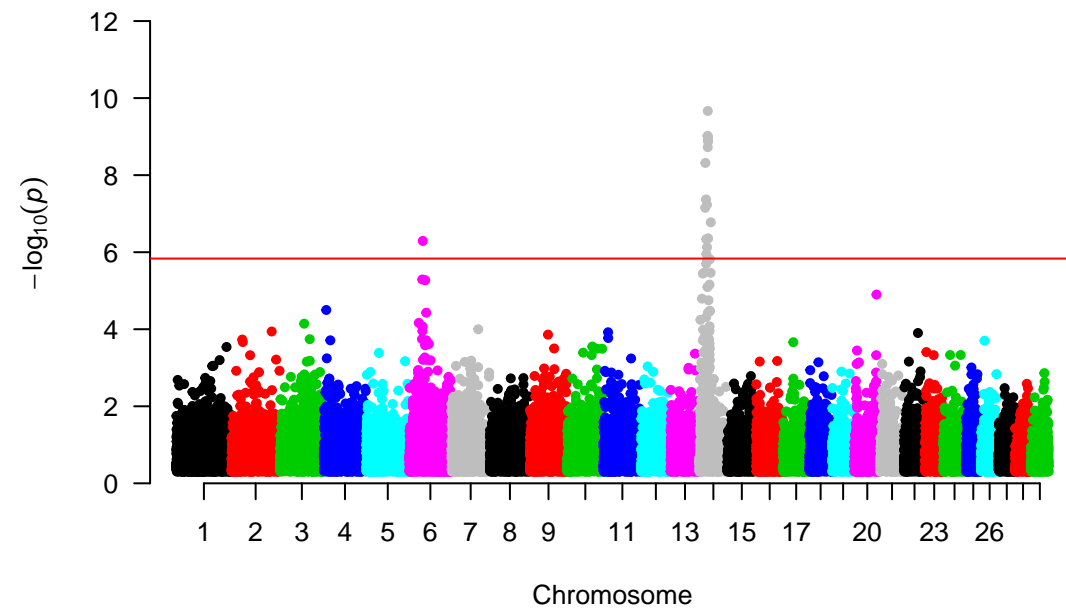**EMA**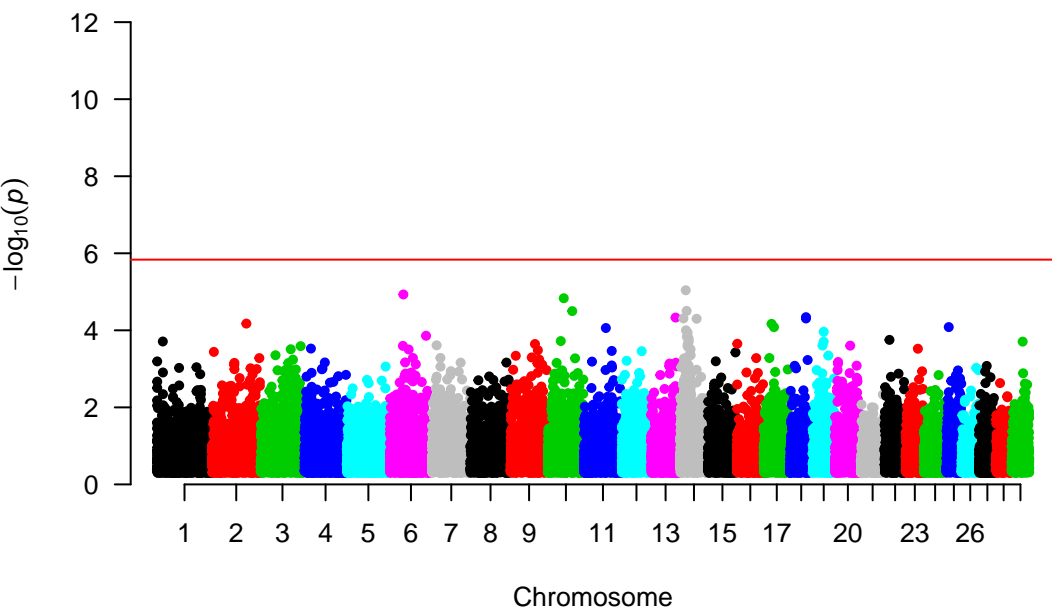**MS**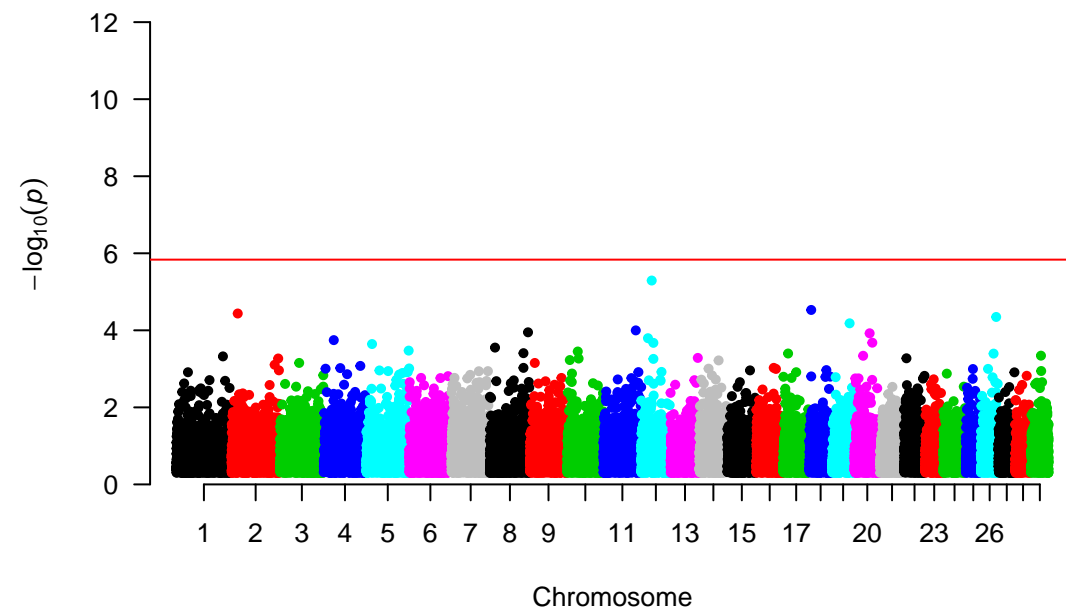

Supplement: Supplementary file 3 — Additional file 3: Figure S2. Manhattan plots of genome-wide association analyses for four carcass traits. This figure provides the log10 p-values of the SNPs analyzed in the genome-wide association analyses for backfat thickness (BT), carcass weight (CW), eye muscle area (EMA), and marbling score (MS) traits. The horizontal lines represent the 5% significance level with a p value threshold of 1.46 × 10^−6^ for backfat thickness (BT), carcass weight (CW), eye muscle area (EMA), and marbling score (MS) traits. [file 12711_2016_283_MOESM3_ESM.pdf]
