# Supplementary material for: Predictive performance of genomic selection methods for carcass traits in Hanwoo beef cattle: impacts of the genetic architecture
Source: Genet Sel Evol. 2017 Jan 4;49:1. doi: 10.1186/s12711-016-0283-0 (PMC5240470; doi:10.1186/s12711-016-0283-0)
Supplement: Supplementary file 2 — Additional file 2: Table S1. Mean square error (SE) of genomic prediction for four carcass traits in Hanwoo beef cattle. This table provides the mean square errors of GBLUP, BayesL and BayesC genomic prediction for backfat thickness (BT), carcass weight (CW), eye muscle area (EMA), and marbling score (MS) traits. [file 12711_2016_283_MOESM2_ESM.pdf]

Trait(Unit)[1]	GBLUP	BayesL	BayesC[a]
BT (mm)	9.606 (0.018)	9.635 (0.018)	9.614 (0.019)
CW (kg)	881.744 (1.70)	883.825 (1.59)	870.119 (2.19)
EMA (cm^2)	53.587 (0.165)	53.759 (0.148)	53.725 (0.156)
lnMS(Score)	0.0990 (0.0003)	0.0994 (0.0003)	0.0996 (0.0004)

[a]In BayesC, the highest accuracy obtained when π (the proportion of markers with no effect) was considered 0.97 (BayesC97) for backfat thickness (BT) and eye muscle area (EMA), 0.99 (BayesC99) for carcass weight (CW) and 0.91 (BayesC91) for marbling score (MS).
