# Supplementary material for: Predictive performance of genomic selection methods for carcass traits in Hanwoo beef cattle: impacts of the genetic architecture
Source: Genet Sel Evol. 2017 Jan 4;49:1. doi: 10.1186/s12711-016-0283-0 (PMC5240470; doi:10.1186/s12711-016-0283-0)
Supplement: Supplementary file 4 — Additional file 4: Table S2. Genomic variance ($\sigma_{g}^{2}$σg2), marker variance explained ($\sigma_{g}^{2} /\sigma_{a}^{2}$σg2/σa2) and genomic heritability ($h_{g}^{2}$hg2) obtained when using fully corrected phenotypes and high-density SNPs with the GBLUP method. Description: This table provides the results for the genomic variance ($\sigma_{g}^{2}$σg2), marker variance explained ($\sigma_{g}^{2} /\sigma_{a}^{2}$σg2/σa2) and genomic heritability ($h_{g}^{2}$hg2) obtained when a high-density chip (777 k) was used to analyze [file 12711_2016_283_MOESM4_ESM.pdf]

**Trait(Unit)**[1]	$\sigma_g^2(SE)$	σ_g^2/σ_a^2	$h_g^2 = h^2\frac{\sigma_g^2}{\sigma_a^2}$
BT (mm)	4.30 (0.84)	0.77	0.38
CW (kg)	306.06 (75.17)	0.97	0.30
EMA (cm^2)	26.59 (4.84)	0.99	0.43
lnMS(Score)	0.065 (0.01)	0.81	0.49

[1]BT, backfat thickness; CW, carcass weight; EMA, eye muscle area; MS, marbling score.
